# A retrospective assessment of COVID-19 vulnerability index indicators and mortality rates pre-COVID-19 (2018–2020) and during COVID-19 (2020–2022) in a health and demographic surveillance site, Soweto, South Africa

**DOI:** 10.1186/s12963-025-00387-9

**Published:** 2025-06-20

**Authors:** Takwanisa Machemedze, Chodziwadziwa Whiteson Kabudula, Jean Juste Harrisson Bashingwa, Beth A. Tippett Barr, Nellie Myburgh, Sana Mahtab, Cleopas Hwinya, Stephen Tollman, Ziyaad Dangor, Shabir A. Madhi

**Affiliations:** 1 South African Medical Research Council, Vaccines and Infectious Diseases Analytical Unit, Faculty of Health Sciences, University of the Witwatersrand, Johannesburg, South Africa; 2 Department of Paediatrics and Child Health, Faculty of Health Sciences, University of the Witwatersrand, Johannesburg, South Africa; 3 Wits Infectious Diseases and Oncology Research Institute, University of the Witwatersrand, Johannesburg, South Africa; 4 South African Medical Research Council/Wits Rural Public Health and Health Transitions Research Unit, Faculty of Health Sciences, University of the Witwatersrand, Johannesburg, South Africa; 5 Nyanja Health Research Institute, Salima, Malawi

**Keywords:** COVID-19, Vulnerability, Risk, Mortality, South Africa, Health and demographic surveillance system

## Abstract

**Background:**

Before COVID-19, knowledge on pandemic vulnerability and mortality in South Africa was largely limited to the context of HIV/AIDS. We evaluated mortality rates and risk of death, prior to and during the COVID-19 pandemic, in relation to an individual’s COVID-19 vulnerability, based on a scoring algorithm developed in South Africa.

**Methods:**

The analysis was undertaken using data from a health and demographic surveillance system (HDSS) in Soweto and Thembelihle, Gauteng, South Africa. Health and demographic population-based data have been collected from the HDSS area since 2018. Using indicators included in a COVID-19 Vulnerability Index, previously developed in South Africa, the current study established a composite COVID-19 vulnerability index, stratified into tertiles. The risk of death pre-COVID-19 (1 January 2018–28 February 2020) and during the COVID-19 period (1 March 2020–31 December 2021) was analysed. A Cox proportional hazard model was used to compare the risk of death between the two time periods. Statistical analyses were conducted using Stata software version 17.

**Results:**

Before COVID-19, overall mortality rates were 8.1 (95% CI 7.6–8.8), 7.0 (95% CI 6.4–7.7) and 6.1 (95% CI 5.5–6.7) per 1000 person-years in the lowest, middle, and highest tertile of vulnerability index, respectively. All cause-mortality across all tertiles more than doubled during the COVID-19 period compared to pre-COVID-19 (15.5 against 7.2). The mortality rates during the COVID-19 era were 17.1 (95% CI 16.3–18.0), 14.5 (95% CI 13.4–15. 7) and 13.7 (95% CI 12.8–14.7) per 1000 person-years in the lowest, middle, and highest tertiles, respectively. Overall, individuals in the highest tertile of COVID-19 vulnerability were at a significantly lower risk of death relative to those in the lowest tertile (aHR 0.9, 95% CI 0.8–1.0, *p* < 0.05). The risk of dying during the COVID-19 period for vulnerable individuals was at least double compared to the pre-COVID-19 period for each of the individual vulnerability indicators.

**Conclusions:**

All-cause mortality during the COVID-19 era was significantly higher than the pre- COVID-19 period, with the increase observed across all vulnerability tertiles. It is important to identify vulnerable individuals and communities during the early stages of a pandemic to inform prioritisation of public health intervention.

**Supplementary Information:**

The online version contains supplementary material available at 10.1186/s12963-025-00387-9.

## Introduction

South Africa, with a complex history of social and economic disparities, experiences significant health burdens among the socially disadvantaged [1]. Prior to the coronavirus disease 2019 (COVID-19) pandemic, non-communicable diseases, such as cardiovascular disease, diabetes, and chronic respiratory conditions, were major contributors to mortality [2, 3]. The human immunodeficiency virus/acquired immunodeficiency syndrome (HIV/AIDS) pandemic has also had a profound impact on the country's health landscape, disproportionately affecting marginalized populations [3]. However, prior to COVID-19, a comprehensive understanding of pandemic vulnerability and its impact on mortality across diverse population groups in South Africa remained limited to HIV/AIDS.

South Africa confirmed its first case of COVID-19 on the 5th of March 2020. In an attempt to dampen the transmission of severe acute respiratory syndrome coronavirius-2 (SARS-CoV-2), that causes COVID-19, the South African government enacted regulations for various non-pharmacological interventions (NPI) from the start of the pandemic (27th March 2020), and fluctuated through to 5th April 2022 when they were completely lifted [4, 5]. The NPI measures included restrictions on non-essential travel with the order to stay-at-home, banning of gatherings and closure of non-essential services [5]. Notably, adherence to the restrictions was low in certain communities due to socio-economic factors, including in Soweto [6–8] which is a low income, peri-urban community where the unemployment rate was 24% in 2011 [9]. The roll out of COVID-19 vaccines to the general population in South Africa started on 17th May 2021, targeted at people older than 60 years of age, and was expanded to the general adult population from 15th July 2021 [5]. Following the third (delta) COVID-19 wave in South Africa, 73% of adults in Gauteng had been infected at least once, which increased to 92% after the subsequent Omicron (BA.1) dominant wave that transpired in November 2022, and at which time only 19% of adults had received at least a single dose of COVID-19 vaccine [10].

During the COVID-19 pandemic, several algorithms were developed or modified to identify vulnerable communities or individuals, with the idea of improving prevention interventions. COVID-19 vulnerability algorithms developed in the United States (US) included the US Centers for Disease Control and Prevention (CDC) Social Vulnerability Index (CDC-SVI) [11], the Pandemic Vulnerability Index (PVI) [12], and the US COVID-19 Community Vulnerability Index (CCVI) [13]. A higher SVI was associated with higher COVID-19 case-fatality rates (i.e. mortality rate per number of cases) [14], but were less accurate in predicting COVID-19 incidence [14, 15].

There is a paucity of data from Africa which have evaluated the vulnerability to SARS-CoV-2 infection and severe COVID-19 at an individual level. A study from the Western Cape province, South Africa, using individual medical history data in the early phase of the pandemic, identified underlying chronic diseases such as diabetes, HIV infection, chronic kidney disease, and older age as risk factors for COVID-19 hospitalization and death [16].

The South Africa COVID-19 vulnerability index (SACVI) aimed at identifying populations most at risk of infection by SARS-CoV-2, and at heighted risk of developing severe COVID-19 [17]. The SACVI algorithm is based on four themes and eight indicators related to mode of transportation to work, access to media, household sanitation services, overcrowding, multi-generational household status, age, and chronic illness. Although the SACVI indicators are applied at an individual level, the intended utility of SACVI was for data spatially aggregated at a population level, to identify communities at highest risk and requiring intervention. The evaluation of the predictive value of the SACVI indicators in relation to SARS-CoV-2 infection and severe COVID-19 at an individual level is only possible through longitudinal population-based surveillance, preferably before and during the course of the COVID-19 pandemic. To our knowledge, we were unable to locate South African studies that examined the SACVI indicators at an individual level, likely due to insufficient data.

We undertook a retrospective analysis of the predictive value of SACVI in relation to mortality rates during the peak of the COVID-19 pandemic period (1 March 2020 to 31 December 2021) and in the pre-pandemic period (1 January 2018 to 28 February 2020) in South Africa using data routinely collected through a demographic and health surveillance system. This study aimed to assess the predictive capacity of the SACVI, including its individual items, in relation to mortality, both before and during the COVID-19 pandemic. The findings provide important insights into the factors influencing mortality risk, particularly in pandemics with epidemiological characteristics similar to COVID-19. These insights can inform targeted interventions to strengthen health systems and address vulnerabilities, thereby improving preparedness and resilience for future public health emergencies.

## Methods

### Data source

We analysed data from a longitudinal population-based cohort from the Soweto and Thembelihle Health and Demographic Surveillance System (SaT-HDSS), that has been continuously collecting data on vital events, health outcomes, and demographic characteristics since 2017. Data for this study were extracted from the SaT-HDSS database for the period, 1st January 2018 to 31st December 2021.

### Study design and setting

The SaT-HDSS includes eight clusters in Soweto and has 91,969 individuals under surveillance. Data collection for the SaT-HDSS was interrupted during the first half of 2020 due to societal restrictions aimed at limiting the spread of SARS-CoV-2 but was resumed in July 2020. The HDSS collects data at an individual level, including new births and in-migration into the area between each survey round, as well as individuals who exit the population due to death or out-migration. Consequently, the HDSS platform has robust population-based denominator data, including the documentation of all deaths in the population. Individual level information is collected from HDSS residents regarding demographic, socioeconomic, and health data, including sex, highest level of education attained and employment status for those 15–64 years of age, and ownership of household assets.

### Study variables

#### Outcome variable

The outcome variable of interest for this study was death, a binary variable indicated by 1 if an HDSS participant died within the study period and 0, otherwise.

#### Independent variables

The main independent variables were SACVI indicators and tertiles of a composite index of the indicators. The SAVCI scoring system indicators [17] are detailed in Table 1, as well as which variables were available for analysis from the HDSS which constituted the COVID-19 vulnerability index (CVI) used in our analysis. The rationale for each indicator provides face validity for the index. The CVI was estimated by adding dummy variables for each indicator, with higher values suggesting greater vulnerability. The study specific CVI was divided into tertiles of vulnerability defined as lowest, middle, and highest.

**Table 1:** Themes and indicators needed to derive the SACVI and their availability in the HDSS

COVID-19 vulnerability themes and indicators	Rationale	Availability
Population		
Employment without ownership of a car	Proxy indicator for risk of shared transport and standing in queues	Yes
No access to internet, radio, and television	Limited access to correct information	Yes
Household services		
No access to water within 200 m of the dwelling	Risk of contracting COVID-19 from neighbours while fetching water	Yes
No access to flush and chemical toilets	Risk of infection from poor sanitation	Yes
Household composition		
Overcrowding status of the household (+ 3 people per room)	Difficult to socially distance from infected household members	Yes
Multigenerational households	Risk of secondary attack rate among the elderly	Yes
Health		
Elderly (+ 60 years)	More likely to die or suffer from a severe COVID-19 infection	Yes
Use of chronic medication	Proxy indicator for health status	No

Data for the indicators in Table 1 were derived from data collected in the HDSS. Some of the variables needed to be tailored to what was collected in the HDSS. For example, the HDSS collects separate data on the employment status of an individual and car ownership by the household. The final variable derived does not necessarily mean that the employed individual owns the car, but that the employed individual lives in a household that owns a car. For the purposes of this study, we adopted multi-generational households definition from elsewhere and defined it as households with at least three or more people, at least one child aged 0–16 years, and at least one person aged 60 years and above [18]. The HDSS did not collect information on the use of chronic medication.

Other independent variables adjusted for in statistical analyses included participant age that was categorised into age groups, sex, highest level of education and employment status for those eligible to work.

The study also used national data on weekly deaths in South Africa that were processed by the South African Medical Research Council (SAMRC) [19, 20]. The SAMRC weekly deaths data were used to compare patterns of deaths in the City of Johannesburg, the municipality in which the HDSS is based, with the HDSS data.

### Data management and statistical analysis

Statistical analysis was performed using Stata software version 17 [21] and the R statistical language, version 4.3.1 [22]. Categorical variables were summarized using frequencies and proportions. Missing individual socio-demographic characteristics were imputed within age groups using the modal characteristic level for the respective age group. To assess the internal consistency reliability of the vulnerability indicators, Cronbach's alpha [23] was calculated. Cronbach's alpha measures the extent to which items within a scale correlate with each other, providing an estimate of the scale's internal consistency. The coefficient of reliability ranges from 0 to 1 and a value of 0.70 or higher is generally considered acceptable, whereas a lower threshold of 0.6 is considered moderate or sufficient [24]. Mortality rates were calculated within the HDSS cohort by dividing the total number of deaths observed within each socio-demographic stratum by the cumulative person-years of observation for that stratum. Survival time was analysed using the Cox proportional hazard (CPH) model [25] to identify factors associated with the risk of dying. The CPH model estimates the hazard ratio, representing the relative risk of an event occurring at any given time, while effectively handling right-censored observations, where the event of interest has not yet occurred for some individuals at the end of the study period. We conducted a post-estimation analysis to determine the effect of COVID-19 vulnerability on changes in all-cause mortality during COVID-19 period using the Stata *lincom* command. Non-overlapping confidence intervals (95%) for mortality rates by categories were used to indicate evidence of significant difference in mortality rates by the categories. In all statistical tests, a p-value less than 0.05 was considered significant.

### Ethical considerations

This study received ethical approval from the University of the Witwatersrand’s Human Ethics Research Committee (HDSS; HREC: Clearance Certificate Number: 170216). Prior to participation, all household representatives provided written informed consent. Participant confidentiality was maintained throughout the study. Data collected were stored securely in a password-protected database.

## Results

### Descriptive statistics

The population structure of the HDSS in 2018 indicated that 67% (58,956/88,234) of the population was older than 18 years of age, 47.2% (41,669/88234) were males, 55.7% (49,158/88234) had only primary-level or no formal education and 35.5% (20,501/57735) of participants aged between 15 and 64 years were unemployed (Table 2). The cumulative study population consisted of 145 695 unique individuals who had ever resided within the HDSS study area. The distribution of vulnerability indicators within this population was as follows: employed but not owning a car (n = 15 687, 10.8%), no access to internet, radio, and television (n = 61 982, 42.5%), no access to water within 200 m of the dwelling (n = 60 928, 41.8%), no access to flush and chemical toilets (n = 18 081, 12.4%), overcrowding status of the household (+ 3 people per room) (n = 50 499, 34.7%), multigenerational household (n = 12 491, 8.6%), living with an elderly (+ 60 years) (n = 52 973, 36.4%). The standardized Cronbach’s alpha coefficient of reliability for the vulnerability items (α = 0.60) was moderately sufficient. The calculated index, consisting of seven items, varied between 0 and 5, an average score of 1.9, a median of 2, interquartile range from 1 to 3. The number of individuals in tertiles of the composite vulnerability index were lower (n = 60 015, 41.2%), middle (n = 38 777, 26.6%), highest (n = 46 903, 32.2%). Results for the vulnerability index tertiles by socio-demographic characteristics are presented in Additional file 1 Table S1.

**Table 2:** Demographic characteristics of study participants at baseline, Soweto, Gauteng, South Africa, 2018

Characteristics	n	%
Age group		
0–14	18,689	21.2
15–24	14,856	16.8
25–39	24,018	27.2
40–59	19,077	21.6
60 +	11,534	13.1
Sex		
Male	41,669	47.2
Female	46,565	52.8
Highest level of education		
None	265	0.3
Primary school (Grade 1–7)	48,893	55.4
Secondary school (Grade 8–11)	37,587	42.6
Matric (Grade 12) and post-matric qualification	1489	1.7
Employment status		
Employed	20,501	35.5
Not employed	37,234	64.5
Total	88,234	100

**Fig. 1:**
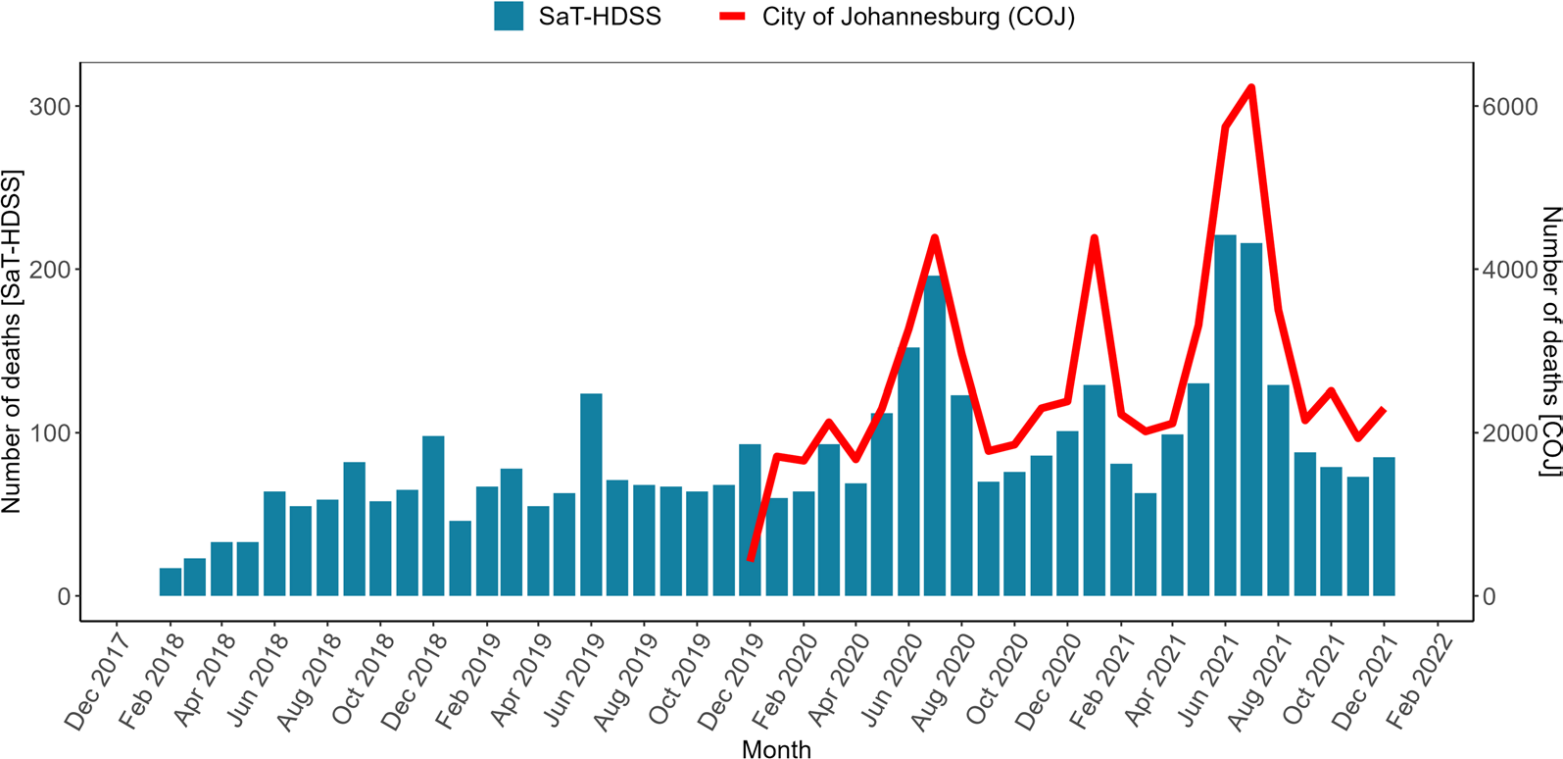
Temporal patterns of deaths

The temporal patterns of deaths observed in the SaT-HDSS site during the COVID-19 period were similar to trends in deaths in the greater Johannesburg municipality area, under which the HDSS falls (Fig. 1).

The overall mortality rate (per 1000 person years) was more than double during the COVID-19 era (15.5, 95% CI 14.9–16.0) compared to pre-COVID-19 (7.2, 95% CI 6.8–7.5) (Table 3). Higher mortality rates were observed across all age groups, with the greatest increase among those aged 60 and above, increasing from 28.3 per 1000 person-years (95% CI 26.4–30.3) in the pre-COVID era to 83.4, (95% CI 79.6–87.3) during the COVID-19 period. Before the COVID-19 pandemic, mortality rates per 1000 person-years were comparable between men (7.1, 95% CI 6.6–7.7) and women (7.2, 95% CI 6.8–7.7). However, during the pandemic, women experienced higher mortality rates (16.3, 95% CI 15.6–17.1) compared to men (14.5, 95% CI 13.7–15.3), with non-overlapping confidence intervals indicating significant difference. Individuals with lower educational attainment (no/primary education) consistently exhibited significantly higher mortality rates compared to those with at least some secondary education. Before the COVID-19 pandemic, mortality rates were 9.1 per 1000 person-years (95% CI 8.6–9.6) for those with no/primary education and 4.7 per 1000 person-years (95% CI 4.3–5.1) for those with at least some secondary education. During the COVID-19 period, these rates increased to 20.2 (95% CI 19.4–21.0) and 8.6 (95% CI 8.0–9.2), respectively. In both periods, the 95% confidence intervals for mortality rates between the two education groups did not overlap, suggesting a significant difference in mortality risk. Mortality rate was generally high among the unemployed compared to the employed, both before COVID-19 (6.8, 95% CI 6.3–7.4, unemployed, 5.5, 95% CI 4.9–6.1, employed) and during COVID-19 (16.3, 95% CI 15.5–17.2, unemployed, 9.3, 95% CI 8.4–10.2, employed).

**Table 3:** Mortality rates before and during the COVID-19 era by demographic and socio-economic factors in Soweto, Gauteng, South Africa (2018–2021)

	Pre- COVID-19 era (January 2018 to February 2020)				COVID-19 era (March 2020 to December 2021)			
	Mortality rate/1000				Mortality rate/1000			
	Deaths	Person-Years	Rate	95% CI	Deaths	Person-Years	Rate	95% CI
Overall	1588	221,143	7.2	(6.8–7.5)	3026	195,788	15.5	(14.9–16.0)
Age group								
0–14	42	84,874	0.5	(0.4, 0.7)	50	57,982	0.9	(0.7, 1.1)
15–24	50	51,429	1.0	(0.7, 1.3)	75	36,156	2.1	(1.7, 2.6)
25–39	235	77,597	3.0	(2.7, 3.4)	337	53,744	6.3	(5.6, 7.0)
40–59	450	59,989	7.5	(6.8, 8.2)	753	43,810	17.2	(16.0, 18.5)
60+	811	28,702	28.3	(26.4, 30.3)	1811	21,715	83.4	(79.6, 87.3)
Sex								
Male	749	105,010	7.1	(6.6, 7.7)	1349	93,210	14.5	(13.7, 15.3)
Female	839	116,133	7.2	(6.8, 7.7)	1677	102,579	16.3	(15.6, 17.1)
Highest level of education								
None/primary	1138	125,210	9.1	(8.6, 9.6)	2344	116,238	20.2	(19.4, 21.0)
At least secondary school	450	95,933	4.7	(4.3, 5.1)	682	79,550	8.6	(8.0, 9.2)
Employment status (15–64 years)								
Not employed	641	94,191	6.8	(6.3, 7.4)	1298	79,545	16.3	(15.5, 17.2)
Employed	286	52,340	5.5	(4.9, 6.1)	433	46,723	9.3	(8.4, 10.2)
COVID-19 Vulnerability Index								
Lowest tertile	748	91,355	8.1	(7.6, 8.8)	1542	90,238	17.1	(16.3, 18.0)
Middle tertile	395	56,333	7.0	(6.4, 7.7)	654	45,073	14.5	(13.4, 15. 7)
Highest tertile	445	73,456	6.1	(5.5, 6.7)	830	60,478	13.7	(12.8, 14.7)

The total number of deaths in each tertile pre-COVID-19 were 748 (lowest), 395 (middle), 445 (highest) and during-COVID-19 were 1542 (lowest), 654 (middle), 830 (highest). Mortality rates (per 1000 person-years) were generally lower for individuals in the highest vulnerability tertile (mortality rate before COVID-19: 6.1, 95% CI 5.5–6.7, mortality rate during COVID-19: 13.7, 95% CI 12.8–14.7) relative to those in the lowest tertile (mortality rate before COVID-19: 8.1, 95% CI 7.6–8.8, mortality rate during COVID-19: 17.1, 95% CI 16.3–18.0). Before COVID-19, mortality rates in the two lowest (8.1, 95% CI 7.6–8.8) and middle (7.0, 95% CI 6.4–7.7) vulnerability tertile were comparable. During the COVID-19 period, mortality rates for the middle (14.5, 95% CI 13.4–15. 7) and highest (13.7, 95% CI 12.8–14.7) vulnerability tertiles were significantly lower than the lowest vulnerability tertile (17.1, 95% CI 16.3–18.0).

The aggregate mortality rates by individual vulnerability indicators are presented in Additional file 1 Table S2.

### Multivariable analysis

The hazard ratios of dying over the period 2018–2021, controlling for selected individual characteristics (sex, age group, level of education), individual COVID-19 vulnerability indicators and COVID-19 period were evaluated first (Table 4). Model 1 includes only the main effects whereas model 2 also includes interaction of tertiles with COVID-19 period. Model 1 is nested in model 2, and a likelihood ratio test was significant suggesting that model 2 was better. The main effects of vulnerability indicators that remain significantly associated with increased risk of dying after interacting each indicator with COVID-19 period included living in a multigenerational household (HR 4.9, 95% CI 2.7–9.2, *p* < 0.001). The main effects for: no access to flush and chemical toilets (HR 0.8, 95% CI 0.71–0.98, *p* = 0.027), overcrowding status of the household (HR 0.8, 95% CI 0.67–0.88, *p* < 0.001) and living in a household with at least one elderly (+ 60 years) person (HR 0.2, 95% CI 0.15–0.19, *p* < 0.001) where all associated with low risk of dying after interacting each indicator with the COVID-19 period. The post analysis of interaction terms show that the risk of dying during the COVID-19 period among those employed and living in a household with no car (aHR 2.0, 95% CI 1.6–2.5, *p* < 0.001), no access to internet, radio, and television (aHR 2.8, 95% CI 1.5–5.1, *p* < 0.001), no access to water within 200 m of the dwelling (aHR 3.5, 95% CI 1.9–6. 6, *p* < 0,001), no access to flush and chemical toilets (aHR 3.8, 95% CI 3.1–4.6, *p* < 0.001), living in an overcrowded household (aHR 2.7, 95% CI 2.3–3.3, *p* < 0.001), living in a multigenerational household (aHR 2.8, 95% CI 1.3–5.7, *p* < 0.001), and being elderly (aHR 3.0, 95% CI 2.6–3.4, *p* < 0.001) were higher compared to the pre-COVID-19 period.

**Table 4:** Hazard ratios of dying by selected baseline characteristics and COVID-19 vulnerability indicators, Soweto, Gauteng, South Africa, 2018–2021

	Model 1			Model 2		
	HR	95% CI	*p*-value	HR	95% CI	*p*-value
Age group						
0–14						
15–24	2.9	[2.2, 3.8]	< 0.001	2.9	[2. 2, 3.8]	< 0.001
25–39	9.5	[7.4, 12.1]	< 0.001	9.5	[7.4, 12.1]	< 0.001
40–59	23.0	[18.2, 29.0]	< 0.001	23.0	[18.3, 29.1]	< 0.001
60+	301.0	[238.8, 379.5]	< 0.001	303.7	[240.8, 383.1]	< 0.001
Sex						
Male						
Female	0.8	[0.7, 0.8]	< 0.001	0.8	[0.7, 0.8]	< 0.001
Highest level of education						
None/primary						
At least secondary school	0.8	[0.8, 0. 9]	0.019	0.8	[0.8, 0. 9]	0.019
COVID-19 period						
Pre-COVID-19						
During COVID-19	2.94	[2.8, 3.1]	< 0.001	3.1	[2.8, 3.5]	< 0.001
Vulnerability indicators						
Population						
Employment without ownership of a car	0.7	[0.6, 0.8]	< 0.001	0.9	[0.8, 1.1]	0.451
No access to internet, radio, and television	1.0	[0.8, 1.3]	0.981	1.1	[0. 7, 1. 8]	0.735
No access to water within 200 m of the dwelling	1.4	[1.1, 1.9]	0.015	1.3	[0.8, 2.2]	0.275
No access to flush and chemical toilets	0.9	[0.9, 1.0]	0.194	0.8	[0.7, 1.0]	0.027
Overcrowding status of the household (+ 3 people per room)	0.7	[0.6, 0.8]	< 0.001	0.8	[0. 7, 0.9]	< 0.001
Multigenerational household	4.5	[3.1, 6.5]	< 0.001	4.9	[2.7, 9.2]	< 0.001
Elderly (+ 60 years)	0.2	[0.2, 0.2]	< 0.001	0.2	[0.2, 0.2]	< 0.001
Interaction × COVID-19						
Employment without ownership of a car × COVID-19 period				0.6	[0.5, 0.8]	< 0.001
No access to internet, radio, and television × COVID-19 period				0.9	[0.5, 1.6]	0.700
No access to water within 200 m of the dwelling × COVID-19 period				1.1	[0.6, 2.1]	0.698
No access to flush and chemical toilets × COVID-19 period				1.2	[1.0, 1.5]	0.070
Overcrowding status of the household (+ 3 people per room) × COVID-19 period				0.9	[0.7, 1.0]	0.086
Multigenerational household × COVID-19 period				0.9	[0.4, 1.8]	0.721
Elderly (+ 60 years) × COVID-19 period				0.9	[0.8, 1.1]	0. 392
N		145 695			145 695	

Table 5 shows hazard ratios of dying over the period 2018–2021, controlling for selected individual characteristics, individual COVID-19 vulnerability tertiles and COVID-19 period. Model 3 includes only the main effects whereas model 4 also includes interaction of tertiles with COVID-19 period. Model 3 is nested in model 4, and a likelihood ratio test was significant suggesting that model 4 was better. The COVID-19 vulnerability tertiles suggest that the hazard of dying for individuals in the highest tertile of vulnerability (aHR 0.9, 95% CI 0.8–1.0, *p* = 0.018) was lower relative to the lowest tertile. The hazard of dying in the middle vulnerability tertile (aHR 1.0, 95% CI 0.9–1.1, *p* = 0.839) was not significantly different to the lowest tertile. The hazards of dying during the COVID-19 period were significantly higher relative to the pre-COVID-19 period (aHR 2.7, 95% CI 2.5–3.0, *p* < 0.001).

**Table 5:** Hazard ratio of dying by selected baseline characteristics and COVID-19 vulnerability index, Soweto, Gauteng, South Africa, 2018–2021

	Model 3			Model 4		
	HR	95% CI	*p*-value	HR	95% CI	*p*-value
Age group						
0–14	Ref					
15–24	2.5	[1.9, 3.4]	< 0.001	2.5	[1.9, 3.4]	< 0.001
25–39	8.0	[6.3, 10.0]	< 0.001	8.0	[6.3, 10.0]	< 0.001
40–59	19.3	[15.6, 23. 9]	< 0.001	19.3	[15. 6,23. 9]	< 0.001
60+	81.5	[66.2,100.4]	< 0.001	81.6	[66.3,100.5]	< 0.001
Sex						
Male	Ref					
Female	0.8	[0.8, 0.9]	< 0.001	0.8	[0.8, 0.9]	< 0.001
Highest level of education						
None/primary	Ref					
At least secondary school	0.8	[0.7, 0.9]	< 0.001	0.8	[0.7, 0.9]	0.001
COVID-19 period						
Pre-COVID-19	Ref					
During COVID-19	2.6	[2. 5, 2.8]	< 0.001	2.7	[2.5, 3.0]	< 0.001
Vulnerability index tertiles						
Lowest	Ref					
Middle	0.9	[0.8, 1.0]	0.009	1.0	[0.9, 1.1]	0.839
Highest	0.9	[0.8, 0.9]	< 0.001	0.9	[0.8,1.0]	0.018
Middle × during COVID-19				0.8	[0.7, 1.0]	0.029
Highest × during COVID-19				1.0	[0.9, 1.2]	0.854
N		145,695			145,695	

The post analysis of interaction effects shows that during the COVID-19 period, the risk of dying among those in the middle tertile (aHR 0.9, 95% CI 0. 8–0.9, *p* < 0,001) and highest tertile (aHR 0.9, 95% CI 0.8–1.0, *p* = 0.003) was lower than those in the lowest tertile of vulnerability. The risk of dying during the COVID-19 period among those in the lowest tertile (aHR 2.7, 95% CI 2.5–3.0, *p* < 0.001), middle tertile (aHR 2.3, 95% CI 2.0–2.6, *p* < 0.001) and highest tertile (aHR 2.7, 95% CI 2.4–3.1, *p* < 0,001) was more than double compared to the pre-COVID-19 period for the respective tertiles.

## Discussion

The current study investigated the utility of selected COVID-19 vulnerability indicators and a COVID-19 vulnerability index to predict mortality and the relative risk of dying pre-pandemic and during the pandemic period among participants from the SaT-HDSS. Mortality rates and hazard of death by categories of a COVID-19 vulnerability index before and during the pandemic differed among people from selected areas from Soweto, Johannesburg, South Africa. The mortality rates were higher during the COVID-19 period compared to the pre-COVID-19 period across selected individual characteristics that includes age, sex, level of education and employment status, COVID-19 vulnerability indicators and categories. Individuals in the highest tertile of COVID-19 vulnerability were at a significantly lower risk of death relative to those in the lowest tertile. The temporal patterns of deaths are consistent with patterns observed in the parent City of Johannesburg municipality [26].

The CVI was meant to identify populations that were most at risk of being affected by COVID-19. The first preferrable outcome measure for evaluating the utility of the COVID-19 vulnerability indicators would have been the incidence of COVID-19 infections. However, it was not possible to know the number of people affected by COVID-19 because of different diagnostic protocols and guidelines. During the initial phase of the pandemic, reverse transcription polymerase chain reaction (RT-PCR) testing for COVID-19 was noted to exhibit a notable rate of false negatives when compared to alternative testing methods [27]. As a result, a significant number of COVID-19 patients were misdiagnosed, making it impossible to know the number of people who suffered COVID-19. The definite measure that has been employed to evaluate the impact of COVID-19 is variation in the number of deaths compared to historical patterns. The current study observed a general increase in the number of deaths during COVID-19 period across all the vulnerability indicators. Several studies have highlighted the relationship between social vulnerability and COVID-19 mortality. In the United States, counties with lower income, lower educational levels, and higher proportions of single-parent households, mobile home residents, and uninsured individuals were associated with higher cumulative COVID-19 death rates [28]. Similarly, in Brazil, regions with high social vulnerability indices experienced higher mortality rates, with significant spatiotemporal clusters of deaths identified in areas with high SVI [29, 30]. It was also observed that not all vulnerability indicators were associated with the hazard of dying. Some of the vulnerability indicators have been criticised for being socioeconomic vulnerabilities rather than health vulnerabilities [31]. Older age, the only health concern indicator that could be used was significantly associated with increased hazard of dying.

Our findings suggest that using a composite index may mask the true effect of individual vulnerability indicators. Five out of seven of the individual vulnerability indicators show significant higher hazards of dying. After combining the indicators into a single index that were divided into tertiles, there was no significant difference in the hazards of death between individuals in lowest and middle tertile of COVID-19 vulnerability. Individuals in the highest tertile of COVID-19 vulnerability were at a significantly lower risk of death relative to those in the lowest tertile. This finding appears contrary to the expectation that those who were considered more vulnerable were more likely to be infected with COVID-19 and potentially more likely to die. This finding, to some extent, can be interpreted to show the limitation of using a composite index to predict morbidity and mortality.

It was also observed that the hazard of dying was not significantly different for some of the individual COVID-19 vulnerability indicators. In the broader context, African countries were considered more vulnerable to COVID-19 and were expected to experience more infections and severe disease/deaths. Several studies have however noted that African countries were less affected by COVID-19 than what was expected [32, 33]. Several hypotheses have been put forward in trying to explain the lower-than-expected COVID-19 related morbidity and mortality in African countries [32]. One of the arguments is that the African population is young and as a result, was at low risk of severe COVID-19 illness. The SaT-HDSS population had a baseline mean and median age of 27 and 25 respectively, and only 4% was aged 65 and above. This can be considered a young population compared to the median age of more than 38 for Europe and the US [33], some of the regions severely affected by COVID-19. It is also argued that older people have health problems that have the potential to increase the risk of severe COVID-19 illness and death. As previously mentioned, the SaT-HDSS population featured a younger age demographic associated with reduced susceptibility to other health-related complications. The CVI derived in the current study might be elevated, however, as it incorporates socio-economic vulnerability indicators that may not necessarily correlate with the risk of death. The use of chronic medication, an indicator for co-morbidities common in older age, was another vulnerability indicator for increased COVID-19 risk [17]. Unfortunately, the current study did not have data on the use of chronic medication. The young population is suggestive of less people using chronic medications and thus less susceptible to severe COVID-19. All these factors may have contributed to the lack of significant differences in the risk of dying by some of the vulnerability indicators.

One possible factor that can be attributed to lower mortality among vulnerable individuals than expected was effective government response. The South African government was quick to enact a national lockdown during the early stages of the pandemic. Schröder et al. [34] evaluated the number of reported COVID-19 cases before and after the first national lockdown and found that the lockdown may have slowed down the number of cases. Slowing down the rate of COVID-19 transmission through the national lockdown also probably helped the public health care system to prepare and cope better with potential COVID-19 patients.

Other explanations for the low COVID-19 mortality in vulnerable African countries include that there are few people in long-term health care facilities which had increased risk of COVID-19 infection, there was cross-protection due to immunity developed from previous infections with other related pathogens such as the influenza, and reduced risk due to genetic factors [32, 33]. The current study could not measure or evaluate these, but they could also have played a part such that there were no disproportionate deaths among the vulnerable as suggested in the literature [32].

This study could not conceptualise all variables needed for creating the SACVI. There was no data on the use of chronic medication, an important measure of health vulnerability. As a result, it was not possible to provide concrete evidence about the utility of the SACVI. Additionally, the study population was not representative of the rest of Soweto or all urban townships in South Africa, and the findings may not be generalisable.

Nonetheless, this study leveraged the strengths of utilizing a robust HDSS dataset, enabling rigorous statistical analysis. The inclusion of both pre-pandemic and pandemic periods allowed for a valuable baseline comparison, facilitating an understanding of how the COVID-19 pandemic altered mortality risk. By focusing on the SACVI indicators, a locally developed vulnerability index, this study offers contextually relevant insights into risk factors for mortality in South Africa. While initially designed for spatial-level vulnerability assessment, analysing the vulnerability indicators and index at the individual level provided deeper insights into the specific factors contributing to individual mortality risk. Furthermore, the use of mortality as the primary outcome, a definitive endpoint, enhances the study's relevance in evaluating pandemic impacts and informing public health preparedness. The findings have significant policy implications, offering actionable insights to strengthen pandemic preparedness and mitigate mortality risk within high-risk groups. Finally, this study contributes valuable contextual evidence from a low- or middle-income country setting, addressing a critical gap in the global understanding of pandemic vulnerability and mortality.

## Conclusion

All-cause mortality during the COVID-19 era was significantly higher than the pre- COVID-19 period. Our study helps inform the understanding of COVID-19 vulnerability at the local level in South Africa and evaluates how surveillance data can complement national data in order to understand disease outbreaks. It is important to identify vulnerable individuals and communities during the early stages of a pandemic to inform prioritisation of public health intervention.

## Supplementary Information

Additional file 1.

## Data Availability

Data for computing mortality indicators by year, age and sex are available from the MRC/Wits Agincourt Research Unit Data Repository (https://data.agincourt.co.za/index.php/catalog/333). Data containing other covariates used in the analysis reported in this manuscript can be accessed through a formal request to the corresponding author.

## Abbreviations

AIDS: Acquired immunodeficiency syndrome
CCVI: COVID-19 community vulnerability index
CVI: COVID-19 vulnerability index
CDC: Centre for Diseases Control and Prevention
COJ: City of Johannesburg
COVID-19: Coronavirus disease of 2019
CPH: Cox proportional hazard
HR: Hazard ratio
HIV: Human immunodeficiency virus
PVI: Pandemic vulnerability index
RSA: Republic of South Africa
SACVI: South Africa COVID-19 vulnerability index
SaT-HDSS: Soweto and Thembelihle Health and Demographic Surveillance System
SVI: Social vulnerability index
