# Supplementary material for: A retrospective assessment of COVID-19 vulnerability index indicators and mortality rates pre-COVID-19 (2018–2020) and during COVID-19 (2020–2022) in a health and demographic surveillance site, Soweto, South Africa
Source: Popul Health Metr. 2025 Jun 20;23(Suppl 2):28. doi: 10.1186/s12963-025-00387-9 (PMC12181823; doi:10.1186/s12963-025-00387-9)
Supplement: Supplementary file 1 — Additional file 1. [file 12963_2025_387_MOESM1_ESM.docx]

# **Supplementary material**

Table S1: Vulnerability tertiles by demographic characteristics

|  | Tertile 1 | | Tertile 2 | | Tertile 2 | | Total | |
| --- | --- | --- | --- | --- | --- | --- | --- | --- |
| **Characteristics** | **n** | **%** | **n** | **%** | **n** | **%** | **n** | **%** |
| Age group |  |  |  |  |  |  |  |  |
| 0-14 | 14461 | 38.5 | 8201 | 21.8 | 14886 | 39.6 | 37548 | 100 |
| 15-24 | 10176 | 42.6 | 5509 | 23.1 | 8204 | 34.3 | 23889 | 100 |
| 25-39 | 15376 | 39 | 13281 | 33.6 | 10817 | 27.4 | 39474 | 100 |
| 40-59 | 13165 | 44.7 | 8543 | 29 | 7770 | 26.4 | 29478 | 100 |
| 60+ | 6837 | 44.7 | 3243 | 21.2 | 5226 | 34.1 | 15306 | 100 |
| Sex |  |  |  |  |  |  |  |  |
| Male | 28589 | 41.4 | 19080 | 27.6 | 21391 | 31 | 69060 | 100 |
| Female | 31426 | 41 | 19697 | 25.7 | 25512 | 33.3 | 76635 | 100 |
| Highest level of education |  |  |  |  |  |  |  |  |
| None | 174 | 43.8 | 109 | 27.5 | 114 | 28.7 | 397 | 100 |
| Primary school (Grade 1-7) | 36358 | 43.8 | 18170 | 21.9 | 28532 | 34.4 | 83060 | 100 |
| Secondary school (Grade 8-11) | 22364 | 37.3 | 19998 | 33.4 | 17518 | 29.3 | 59880 | 100 |
| Matric (Grade 12) and post-matric qualification | 1119 | 47.5 | 500 | 21.2 | 739 | 31.3 | 2358 | 100 |
| Employment status |  |  |  |  |  |  |  |  |
| Employed | 11135 | 33.3 | 12421 | 37.2 | 9868 | 29.5 | 33424 | 100 |
| Not employed | 48880 | 43.5 | 26356 | 23.5 | 37035 | 33 | 112271 | 100 |
| Total | 60015 | 41.2 | 38777 | 26.6 | 46903 | 32.2 | 145695 | 100 |

Table S2: Mortality rates before and during the COVID-19 era by categories of individual and household vulnerability indicators in Soweto, Gauteng, South Africa (2018–2021)

|  | **2018 - 2020** | | | | **2020-2021** | | | |
| --- | --- | --- | --- | --- | --- | --- | --- | --- |
|  |  |  | **Mortality rate/1000** | |  |  | **Mortality rate/1000** | |
|  | **Deaths** | **Person-Years** | **Rate** | **95% CI** | **Deaths** | **Person-Years** | **Rate** | **95% CI** |
| Population |  |  |  |  |  |  |  |  |
| *Employment without ownership of a car* | | | | | |  |  |  |
| No | 1463 | 196126 | 7.5 | (7.1, 7.9) | 2812 | 170864 | 16.5 | (15.9, 17.1) |
| Yes | 125 | 25016 | 5.0 | (4.2, 6.0) | 214 | 24924 | 8.6 | (7.5, 9.8) |
| *No access to internet, radio, and television* | | | | | | |  |  |
| No | 906 | 129448 | 7.0 | (6.6, 7.5) | 1895 | 127837 | 14.8 | (14.2, 15.5) |
| Yes | 682 | 91695 | 7.4 | (6.9, 8.0) | 1131 | 67951 | 16.6 | (15.7, 17.6) |
| Household services | |  |  |  |  |  |  |  |
| *No access to water within 200m of the dwelling* | | | | | | | |  |
| No | 919 | 131050 | 7.0 | (6.6, 7.5) | 1917 | 129452 | 14.8 | (14.2, 15.5) |
| Yes | 669 | 90093 | 7.4 | (6.9, 8.0) | 1109 | 66337 | 16.7 | (15.8, 17.7) |
| *No access to flush and chemical toilets* | | | | | |  |  |  |
| No | 1410 | 192658 | 7.3 | (6.9, 7.7) | 2587 | 167787 | 15.4 | (14.8, 16.0) |
| Yes | 178 | 28485 | 6.2 | (5.4, 7.2) | 439 | 28001 | 15.7 | (14.3, 17.2) |
| Household composition | | |  |  |  |  |  |  |
| *Overcrowding status of the household (+3 people per room)* | | | | | | | | |
| No | 1199 | 142869 | 8.4 | (7.9, 8.9) | 2373 | 128154 | 18.5 | (17.8, 19.3) |
| Yes | 389 | 78274 | 5.0 | (4.5, 5.5) | 653 | 67634 | 9.7 | (8.9, 10.4) |
| *Co-residence of 60+ & children (0-14)* | | | | | |  |  |  |
| No | 1577 | 196391 | 8.0 | (7.6, 8.4) | 3004 | 174139 | 17.3 | (16.6, 17.9) |
| Yes | 11 | 24752 | 0.4 | (0.2, 0.8) | 22 | 21649 | 1.0 | (0.7, 1.5) |
| *Co-residence of 60+ & youth (15-24)* | | | | |  |  |  |  |
| No | 1557 | 205346 | 7.6 | (7.2, 8.0) | 2996 | 181299 | 16.5 | (15.9, 17.1) |
| Yes | 31 | 15797 | 2.0 | (1.4, 2.8) | 30 | 14490 | 2.1 | (1.4, 3.0) |
| *Co-residence of 60+ & early adulthood (25-39)* | | | | | | |  |  |
| No | 1490 | 200497 | 7.4 | (7.1, 7.8) | 2871 | 176683 | 16.2 | (15.7, 16.9) |
| Yes | 98 | 20646 | 4.7 | (3.9, 5.8) | 155 | 19105 | 8.1 | (6.9, 9.5) |
| *Co-residence of 60+ & middle adulthood (40-59)* | | | | | | | |  |
| No | 1411 | 204309 | 6.9 | (6.6, 7.3) | 2750 | 181829 | 15.1 | (14.6, 15.7) |
| Yes | 177 | 16834 | 10.5 | (9.1, 12.2) | 276 | 13959 | 19.8 | (17.6, 22.2) |
| Health |  |  |  |  |  |  |  |  |
| *Elderly (+60 years)* | |  |  |  |  |  |  |  |
| No | 460 | 122568 | 3.8 | (3.4, 4.1) | 732 | 106631 | 6.9 | (6.4, 7.4) |
| Yes | 1128 | 98575 | 11.4 | (10.8, 12.1) | 2294 | 89158 | 25.7 | (24.7, 26.8) |
